# Supplementary material for: Endemic vascular plants provide reliable indicators for mapping seasonally dry tropical forests
Source: PLoS One. 2025 Dec 9;20(12):e0337886. doi: 10.1371/journal.pone.0337886 (PMC12688124; doi:10.1371/journal.pone.0337886)
Supplement: S1 Table — (DOCX) [file pone.0337886.s001.docx]

**SUPPLEMENTARY MATERIAL**

**Are endemic species a suitable surrogate for delineating the geographical distribution of the tropical dry forest?**

**Mayra Flores-Tolentino, José Luis Villaseñor, Enrique Ortíz, David A. Prieto-Torres, Guillermo Ibarra-Manríquez**

**PLOS One**

**S1 Table.** List of modeled species, modeling metrics, and model evaluation metrics, indicating records to each species present in the Seasonally Dry Tropical Forest (STDF). Unique: number of records after apply the filter of 5 km. Type partition of records for modeling: BOOT: Random bootstrap partition (species with 5 to 20 records) and KFOLD: Random partition in k-fold cross-validation (species with 20 or more record). Assessing the performance of SDM: AUC: Area Under the Curve, TSS: True Skill Statistic and Boyce index.

| **Families/Species** | **Records** |  |  | **Partition** | **AUC** | **Kappa** | **TSS** | **Boyce** |
| --- | --- | --- | --- | --- | --- | --- | --- | --- |
|  | **Total** | **STDF (%)** | **Unique** |  |  |  |  |  |
| **Acanthaceae** |  |  |  |  |  |  |  |  |
| *Anisacanthus andersonii* | 39 | 20 (51) | 6 | BOOT | 1.0 | 1.0 | 1.0 | 1.0 |
| *Dicliptera haenkeana* | 54 | 48 (89) | 5 | BOOT | 1.0 | 1.0 | 1.0 | NA |
| *Elytraria mexicana* | 78 | 63 (81) | 5 | BOOT | 1.0 | 1.0 | 1.0 | NA |
| *Holographis pallida* | 16 | 14 (88) | 8 | BOOT | 1.0 | 1.0 | 1.0 | 1.0 |
| *Justicia zopilotensis* | 37 | 22 (59) | 7 | BOOT | 1.0 | 1.0 | 1.0 | 1.0 |
| *Tetramerium diffusum* | 40 | 32 (80) | 7 | BOOT | 1.0 | 1.0 | 1.0 | 1.0 |
| *Tetramerium glutinosum* | 57 | 44 (77) | 27 | KFOLD | 1.0 | 0.8 | 0.8 | 0.6 |
| *Tetramerium langlassei* | 20 | 14 (70) | 10 | BOOT | 0.9 | 0.7 | 0.7 | 0.4 |
| *Tetramerium ochoterenae* | 55 | 45 (82) | 18 | BOOT | 1.0 | 1.0 | 1.0 | 1.0 |
| **Amaranthaceae** |  |  |  |  |  |  |  |  |
| *Alternanthera stellata* | 83 | 60 (72) | 29 | KFOLD | 0.9 | 0.0 | 0.0 | 0.8 |
| *Iresine hartmanii* | 100 | 63 (63) | 12 | BOOT | 0.6 | 0.4 | 0.4 | 0.3 |
| **Apocynaceae** |  |  |  |  |  |  |  |  |
| *Cascabela pinifolia* | 69 | 61 (88) | 15 | BOOT | 1.0 | 1.0 | 1.0 | 1.0 |
| *Echites asperoglottis* | 81 | 78 (96) | 6 | BOOT | 1.0 | 1.0 | 1.0 | 1.0 |
| *Matelea petiolaris* | 67 | 39 (58) | 19 | BOOT | 1.0 | 0.9 | 0.9 | 0.9 |
| *Suberogerens cyclophylla* | 102 | 65 (64) | 26 | KFOLD | 0.9 | 0.5 | 0.5 | 0.7 |
| **Aristolochiaceae** |  |  |  |  |  |  |  |  |
| *Aristolochia cardiantha* | 12 | 11 (92) | 5 | BOOT | 1.0 | 1.0 | 1.0 | NA |
| *Aristolochia mycteria* | 21 | 13 (62) | 7 | BOOT | 1.0 | 1.0 | 1.0 | 1.0 |
| **Asparagaceae** |  |  |  |  |  |  |  |  |
| *Agave petrophila* | 35 | 29 (83) | 7 | BOOT | 1.0 | 1.0 | 1.0 | 1.0 |
| *Beaucarnea goldmanii* | 83 | 51 (61) | 7 | BOOT | 0.9 | 0.8 | 0.8 | 0.8 |
| *Beaucarnea recurvata* | 209 | 134 (64) | 19 | BOOT | 1.0 | 1.0 | 1.0 | 1.0 |
| *Echeandia breedlovei* | 23 | 16 (79) | 6 | BOOT | 1.0 | 1.0 | 1.0 | 1.0 |
| *Furcraea macdougallii* | 54 | 27 (50) | 11 | BOOT | 1.0 | 1.0 | 1.0 | 1.0 |
| **Asteraceae** |  |  |  |  |  |  |  |  |
| *Acourtia lepidopoda* | 28 | 21 (75) | 5 | BOOT | 1.0 | 1.0 | 1.0 | NA |
| *Aldama michoacana* | 78 | 64 (82) | 16 | BOOT | 1.0 | 1.0 | 1.0 | 1.0 |
| *Bidens mexicana* | 81 | 42 (52) | 11 | BOOT | 0.8 | 0.7 | 0.7 | 0.7 |
| *Bidens sambucifolia* | 60 | 56 (93) | 9 | BOOT | 1.0 | 0.5 | 0.5 | 1.0 |
| *Cosmos pacificus* | 51 | 41 (80) | 7 | BOOT | 1.0 | 1.0 | 1.0 | 1.0 |
| *Cymophora accedens* | 36 | 32 (89) | 17 | BOOT | 1.0 | 1.0 | 1.0 | 1.0 |
| *Cymophora pringlei* | 67 | 57 (85) | 10 | BOOT | 1.0 | 1.0 | 1.0 | 1.0 |
| *Eremosis villaregalis* | 41 | 27 (66) | 11 | BOOT | 1.0 | 1.0 | 1.0 | 1.0 |
| *Flaveria sonorensis* | 19 | 18 (95) | 5 | BOOT | 1.0 | 1.0 | 1.0 | NA |
| *Heliopsis parviceps* | 9 | 7 (78) | 5 | BOOT | 1.0 | 1.0 | 1.0 | NA |
| *Hofmeisteria standleyi* | 30 | 16 (53) | 7 | BOOT | 0.5 | 0.3 | 0.3 | 0.5 |
| *Lagascea aurea* | 86 | 86 (100) | 29 | KFOLD | 1.0 | 0.9 | 0.9 | 0.7 |
| *Lagascea palmeri* | 55 | 39 (71) | 7 | BOOT | 1.0 | 1.0 | 1.0 | 1.0 |
| *Melampodium nutans* | 115 | 61 (53) | 11 | BOOT | 1.0 | 0.5 | 0.5 | 1.0 |
| *Melampodium pilosum* | 162 | 129 (80) | 5 | BOOT | 1.0 | 1.0 | 1.0 | NA |
| *Otopappus epaleaceus* | 225 | 156 (69) | 10 | BOOT | 1.0 | 1.0 | 1.0 | 1.0 |
| *Pectis exserta* | 94 | 50 (53) | 9 | BOOT | 0.9 | 0.8 | 0.8 | 0.8 |
| *Pectis holochaeta* | 41 | 39 (95) | 17 | BOOT | 1.0 | 0.5 | 0.5 | 1.0 |
| *Pectis luckoviae* | 25 | 21 (84) | 7 | BOOT | 1.0 | 1.0 | 1.0 | 1.0 |
| *Perityle cuneata* | 221 | 112 (51) | 5 | BOOT | 1.0 | 0.5 | 0.5 | NA |
| *Pittocaulon filare* | 49 | 35 (71) | 8 | BOOT | 1.0 | 1.0 | 1.0 | 1.0 |
| *Porophyllum calcicola* | 124 | 98 (79) | 9 | BOOT | 1.0 | 1.0 | 1.0 | 1.0 |
| *Porophyllum pringlei* | 81 | 47 (58) | 25 | KFOLD | 0.8 | 0.4 | 0.4 | 0.4 |
| *Sclerocarpus multifidus* | 63 | 56 (89) | 7 | BOOT | 1.0 | 1.0 | 1.0 | 1.0 |
| *Sclerocarpus papposus* | 112 | 87 (78) | 30 | KFOLD | 1.0 | 0.8 | 0.8 | 0.7 |
| *Trixis calcicola* | 13 | 12 (92) | 6 | BOOT | 1.0 | 1.0 | 1.0 | NA |
| *Zinnia microglossa* | 35 | 26 (74) | 10 | BOOT | 1.0 | 1.0 | 1.0 | 1.0 |
| **Boraginaceae** |  |  |  |  |  |  |  |  |
| *Cordia globulifera* | 60 | 46 (77) | 5 | BOOT | 1.0 | 1.0 | 1.0 | NA |
| *Cordia igualensis* | 37 | 27 (73) | 11 | BOOT | 1.0 | 1.0 | 1.0 | 1.0 |
| *Cordia morelosana* | 290 | 223 (77) | 39 | KFOLD | 0.9 | 0.3 | 0.3 | 0.2 |
| *Cordia seleriana* | 237 | 156 (66) | 37 | KFOLD | 1.0 | 0.8 | 0.8 | 0.6 |
| *Pholisma culiacana* | 21 | 13 (62) | 6 | BOOT | 1.0 | 1.0 | 1.0 | 1.0 |
| **Bromeliaceae** |  |  |  |  |  |  |  |  |
| *Bromelia palmeri* | 76 | 43 (57) | 17 | BOOT | 0.9 | 0.6 | 0.6 | 0.4 |
| *Hechtia sphaeroblasta* | 23 | 14 (61) | 8 | BOOT | 0.5 | 0.5 | 0.5 | 1.0 |
| *Tillandsia intermedia* | 85 | 60 (71) | 12 | BOOT | 1.0 | 1.0 | 1.0 | 1.0 |
| **Burseraceae** |  |  |  |  |  |  |  |  |
| *Bursera bolivarii* | 251 | 209 (83) | 23 | KFOLD | 1.0 | 1.0 | 1.0 | 1.0 |
| *Bursera cinerea* | 242 | 170 (70) | 33 | KFOLD | 0.9 | 0.0 | 0.0 | 0.3 |
| *Bursera confusa* | 175 | 111 (63) | 36 | KFOLD | 0.8 | 0.1 | 0.1 | 0.6 |
| *Bursera coyucensis* | 195 | 188 (96) | 27 | KFOLD | 1.0 | 0.5 | 0.5 | 0.8 |
| *Bursera crenata* | 220 | 220 (100) | 9 | BOOT | 1.0 | 1.0 | 1.0 | 1.0 |
| *Bursera discolor* | 256 | 148 (58) | 29 | KFOLD | 1.0 | 0.9 | 0.9 | 0.4 |
| *Bursera infernidialis* | 87 | 87 (100) | 9 | BOOT | 1.0 | 1.0 | 1.0 | 1.0 |
| *Bursera isthmica* | 38 | 28 (74) | 6 | BOOT | 1.0 | 1.0 | 1.0 | 1.0 |
| *Bursera kerberi* | 412 | 263 (64) | 56 | KFOLD | 1.0 | 0.4 | 0.4 | 0.1 |
| *Bursera laurihuertae* | 149 | 90 (60) | 9 | BOOT | 1.0 | 1.0 | 1.0 | 1.0 |
| *Bursera mirandae* | 213 | 135 (63) | 17 | BOOT | 1.0 | 0.5 | 0.5 | 1.0 |
| *Bursera multifolia* | 79 | 66 (84) | 6 | BOOT | 1.0 | 1.0 | 1.0 | 1.0 |
| *Bursera paradoxa* | 113 | 108 (96) | 20 | KFOLD | 1.0 | 1.0 | 1.0 | 1.0 |
| *Bursera ribana* | 50 | 39 (78) | 5 | BOOT | 1.0 | 1.0 | 1.0 | NA |
| *Bursera sarukhanii* | 175 | 157 (90) | 9 | BOOT | 1.0 | 1.0 | 1.0 | 1.0 |
| *Bursera staphyleoides* | 26 | 22 (85) | 7 | BOOT | 1.0 | 0.5 | 0.5 | 1.0 |
| *Bursera trifoliolata* | 103 | 99 (96) | 7 | BOOT | 1.0 | 0.5 | 0.5 | 1.0 |
| *Bursera trimera* | 232 | 211 (91) | 7 | BOOT | 1.0 | 1.0 | 1.0 | 1.0 |
| *Bursera vejar-vazquezii* | 161 | 122 (76) | 7 | BOOT | 0.9 | 0.8 | 0.8 | 1.0 |
| **Cactaceae** |  |  |  |  |  |  |  |  |
| *Acanthocereus maculatus* | 8 | 8 (100) | 5 | BOOT | 1.0 | 1.0 | 1.0 | NA |
| *Acanthocereus rosei* | 13 | 13(100) | 5 | BOOT | 1.0 | 1.0 | 1.0 | NA |
| *Ferocactus pottsii* | 13 | 10 (77) | 5 | BOOT | 0.0 | 0.0 | 0.0 | NA |
| *Mammillaria beneckei* | 30 | 30 (100) | 14 | BOOT | 1.0 | 0.9 | 0.9 | 0.9 |
| *Mammillaria polyedra* | 20 | 14 (70) | 9 | BOOT | 1.0 | 1.0 | 1.0 | 1.0 |
| *Opuntia excelsa* | 122 | 80 (66) | 7 | BOOT | 1.0 | 1.0 | 1.0 | 1.0 |
| *Opuntia tehuantepecana* | 25 | 18 (72) | 6 | BOOT | 1.0 | 1.0 | 1.0 | 1.0 |
| *Pereskiopsis kellermanii* | 36 | 22 (61) | 9 | BOOT | 1.0 | 1.0 | 1.0 | 1.0 |
| *Pilosocereus purpusii* | 72 | 48 (67) | 19 | BOOT | 1.0 | 0.8 | 0.8 | 0.6 |
| *Pilosocereus quadricentralis* | 16 | 12 (75) | 5 | BOOT | 1.0 | 1.0 | 1.0 | NA |
| *Stenocereus beneckei* | 46 | 32 (70) | 18 | BOOT | 0.9 | 0.4 | 0.4 | 0.6 |
| *Stenocereus fricii* | 17 | 16 (94) | 8 | BOOT | 1.0 | 0.5 | 0.5 | 1.0 |
| *Stenocereus kerberi* | 31 | 18 (58) | 6 | BOOT | 0.9 | 0.5 | 0.5 | 1.0 |
| *Stenocereus standleyi* | 51 | 37 (73) | 9 | BOOT | 1.0 | 1.0 | 1.0 | 1.0 |
| **Celastraceae** |  |  |  |  |  |  |  |  |
| *Crossopetalum managuatillo* | 62 | 62 (100) | 27 | KFOLD | 1.0 | 0.9 | 0.9 | 0.6 |
| **Convolvulaceae** |  |  |  |  |  |  |  |  |
| *Ipomoea chamelana* | 34 | 31 (91) | 13 | BOOT | 1.0 | 1.0 | 1.0 | NA |
| *Ipomoea pseudoracemosa* | 21 | 19 (90) | 12 | BOOT | 1.0 | 1.0 | 1.0 | 1.0 |
| **Cucurbitaceae** |  |  |  |  |  |  |  |  |
| *Cyclanthera longisepala* | 80 | 50 (63) | 8 | BOOT | 0.6 | 0.3 | 0.3 | NA |
| *Cyclanthera micrantha* | 10 | 10 (100) | 6 | BOOT | 1.0 | 1.0 | 1.0 | 1.0 |
| *Ibervillea fusiformis* | 94 | 78 (83) | 40 | KFOLD | 0.9 | 0.6 | 0.6 | 0.4 |
| *Ibervillea hypoleuca* | 84 | 53 (63) | 5 | BOOT | 1.0 | 1.0 | 1.0 | NA |
| *Ibervillea maxima* | 66 | 33 (50) | 5 | BOOT | 1.0 | 1.0 | 1.0 | NA |
| *Sechiopsis tetraptera* | 120 | 85 (71) | 26 | KFOLD | 1.0 | 0.8 | 0.8 | 0.2 |
| *Sicyos sinaloae* | 11 | 11 (100) | 9 | BOOT | 0.9 | 0.7 | 0.7 | 1.0 |
| **Cyperaceae** |  |  |  |  |  |  |  |  |
| *Cyperus sordidus* | 57 | 29 (51) | 9 | BOOT | 1.0 | 1.0 | 1.0 | 1.0 |
| **Dioscoreaceae** |  |  |  |  |  |  |  |  |
| *Dioscorea gallegosi* | 47 | 34 (72) | 16 | BOOT | 0.9 | 0.8 | 0.8 | 0.2 |
| *Dioscorea guerrerensis* | 51 | 26 (51) | 8 | BOOT | 1.0 | 1.0 | 1.0 | 1.0 |
| *Dioscorea morelosana* | 55 | 29 (53) | 14 | BOOT | 1.0 | 1.0 | 1.0 | 1.0 |
| *Dioscorea palmeri* | 30 | 21 (70) | 7 | BOOT | 1.0 | 1.0 | 1.0 | 1.0 |
| *Dioscorea platycolpota* | 44 | 24 (55) | 11 | BOOT | 1.0 | 1.0 | 1.0 | 1.0 |
| **Ebenaceae** |  |  |  |  |  |  |  |  |
| *Diospyros sonorae* | 99 | 63 (64) | 6 | BOOT | 1.0 | 1.0 | 1.0 | 1.0 |
| **Euphorbiaceae** |  |  |  |  |  |  |  |  |
| *Bernardia spongiosa* | 54 | 43 (80) | 6 | BOOT | 1.0 | 1.0 | 1.0 | 1.0 |
| *Cnidoscolus sinaloensis* | 31 | 21 (68) | 5 | BOOT | 1.0 | 1.0 | 1.0 | NA |
| *Croton balsensis* | 18 | 15 (83) | 8 | BOOT | 1.0 | 1.0 | 1.0 | 1.0 |
| *Croton culiacanensis* | 46 | 32 (70) | 10 | BOOT | 1.0 | 1.0 | 1.0 | 1.0 |
| *Croton flavescens* | 412 | 323 (78) | 43 | KFOLD | 0.9 | 0.5 | 0.5 | 0.2 |
| *Croton lindquistii* | 13 | 13 (100) | 10 | BOOT | 1.0 | 1.0 | 1.0 | 1.0 |
| *Croton roxanae* | 112 | 59 (53) | 5 | BOOT | 1.0 | 1.0 | 1.0 | NA |
| *Croton suberosus* | 373 | 265 (71) | 12 | BOOT | 0.9 | 0.6 | 0.6 | 1.0 |
| *Ditaxis manzanilloana* | 120 | 81 (68) | 5 | BOOT | 1.0 | 1.0 | 1.0 | NA |
| *Euphorbia apatzingana* | 43 | 40 (93) | 20 | KFOLD | 1.0 | 1.0 | 1.0 | 1.0 |
| *Euphorbia linguiformis* | 34 | 34 (100) | 10 | BOOT | 1.0 | 1.0 | 1.0 | 1.0 |
| *Euphorbia umbellulata* | 106 | 70 (66) | 6 | BOOT | 1.0 | 1.0 | 1.0 | 1.0 |
| *Jatropha alamanii* | 80 | 54 (68) | 14 | BOOT | 1.0 | 1.0 | 1.0 | 1.0 |
| *Jatropha andrieuxii* | 32 | 22 (69) | 5 | BOOT | 1.0 | 1.0 | 1.0 | NA |
| *Jatropha malacophylla* | 147 | 96 (65) | 27 | KFOLD | 0.8 | 0.5 | 0.5 | 0.4 |
| *Jatropha mcvaughii* | 31 | 21 (68) | 11 | BOOT | 0.7 | 0.5 | 0.5 | 0.7 |
| *Jatropha pseudocurcas* | 36 | 23 (64) | 10 | BOOT | 1.0 | 1.0 | 1.0 | 1.0 |
| *Jatropha websteri* | 11 | 11 (100) | 7 | BOOT | 0.9 | 0.8 | 0.8 | 1.0 |
| *Manihot caudata* | 145 | 98 (68) | 16 | BOOT | 0.7 | 0.3 | 0.3 | 0.5 |
| *Manihot crassisepala* | 39 | 20 (51) | 5 | BOOT | 1.0 | 1.0 | 1.0 | NA |
| *Manihot michaelis* | 46 | 31 (67) | 13 | BOOT | 1.0 | 1.0 | 1.0 | 1.0 |
| *Manihot tomatophylla* | 68 | 56 (82) | 16 | BOOT | 1.0 | 1.0 | 1.0 | 1.0 |
| *Pleradenophora lottiae* | 41 | 27 (66) | 10 | BOOT | 1.0 | 1.0 | 1.0 | 1.0 |
| **Fabaceae** |  |  |  |  |  |  |  |  |
| *Acaciella rosei* | 95 | 88 (93) | 10 | BOOT | 0.9 | 0.8 | 0.8 | 0.8 |
| *Bauhinia subrotundifolia* | 124 | 69 (56) | 20 | KFOLD | 1.0 | 0.8 | 0.8 | 0.3 |
| *Brongniartia alamosana* | 111 | 84 (76) | 31 | KFOLD | 0.9 | 0.3 | 0.3 | 0.5 |
| *Conzattia sericea* | 33 | 27 (82) | 6 | BOOT | 1.0 | 1.0 | 1.0 | 1.0 |
| *Coursetia polyphylla* | 71 | 60 (85) | 12 | BOOT | 1.0 | 1.0 | 1.0 | 1.0 |
| *Desmanthus interior* | 36 | 23 (64) | 9 | BOOT | 1.0 | 1.0 | 1.0 | 1.0 |
| *Erythrina oliviae* | 22 | 15 (68) | 6 | BOOT | 1.0 | 1.0 | 1.0 | 1.0 |
| *Erythrostemon acapulcensis* | 41 | 32 (78) | 7 | BOOT | 0.9 | 0.8 | 0.8 | 1.0 |
| *Erythrostemon oyamae* | 42 | 39 (93) | 12 | BOOT | 1.0 | 1.0 | 1.0 | 1.0 |
| *Erythrostemon standleyi* | 15 | 8 (53) | 6 | BOOT | 0.5 | 0.0 | 0.0 | NA |
| *Heteroflorum sclerocarpum* | 81 | 42 (52) | 8 | BOOT | 1.0 | 1.0 | 1.0 | 1.0 |
| *Indigofera platycarpa* | 180 | 109 (61) | 7 | BOOT | 1.0 | 1.0 | 1.0 | 1.0 |
| *Leucaena matudae* | 22 | 13 (59) | 9 | BOOT | 1.0 | 1.0 | 1.0 | 1.0 |
| *Lonchocarpus balsensis* | 60 | 60 (100) | 32 | KFOLD | 1.0 | 0.8 | 0.8 | 0.5 |
| *Lonchocarpus huetamoensis* | 39 | 35 (90) | 14 | BOOT | 1.0 | 1.0 | 1.0 | 1.0 |
| *Lonchocarpus longipedunculatus* | 32 | 30 (94) | 17 | BOOT | 1.0 | 1.0 | 1.0 | 1.0 |
| *Lonchocarpus magallanesii* | 41 | 41 (100) | 11 | BOOT | 1.0 | 1.0 | 1.0 | 1.0 |
| *Lonchocarpus minor* | 28 | 21 (75) | 7 | BOOT | 1.0 | 1.0 | 1.0 | 1.0 |
| *Lonchocarpus obovatus* | 89 | 51 (57) | 16 | BOOT | 1.0 | 1.0 | 1.0 | 1.0 |
| *Lonchocarpus parviflorus* | 97 | 66 (68) | 6 | BOOT | 0.5 | 0.3 | 0.3 | 1.0 |
| *Lonchocarpus schubertiae* | 85 | 81 (95) | 40 | KFOLD | 1.0 | 1.0 | 1.0 | 1.0 |
| *Lonchocarpus sinaloensis* | 20 | 11 (55) | 8 | BOOT | 1.0 | 1.0 | 1.0 | 1.0 |
| *Macroptilium pedatum* | 12 | 12 (100) | 7 | BOOT | 1.0 | 1.0 | 1.0 | 1.0 |
| *Marina holwayi* | 37 | 29 (78) | 14 | BOOT | 1.0 | 1.0 | 1.0 | 1.0 |
| *Marina palmeri* | 32 | 27 (84) | 9 | BOOT | 1.0 | 1.0 | 1.0 | 1.0 |
| *Microlobius foetidus* | 104 | 85 (82) | 39 | KFOLD | 1.0 | 0.9 | 0.9 | 0.9 |
| *Mimosa egregia* | 75 | 72 (96) | 11 | BOOT | 1.0 | 1.0 | 1.0 | 1.0 |
| *Mimosa moniliformis* | 32 | 20 (63) | 8 | BOOT | 0.9 | 0.8 | 0.8 | 1.0 |
| *Mimosa psilocarpa* | 76 | 43 (57) | 16 | BOOT | 1.0 | 0.5 | 0.5 | 0.8 |
| *Mimosa rosei* | 170 | 140 (82) | 8 | BOOT | 0.6 | 0.5 | 0.5 | 0.8 |
| *Phaseolus macvaughii* | 49 | 39 (80) | 12 | BOOT | 0.9 | 0.8 | 0.8 | 1.0 |
| *Piscidia mollis* | 89 | 58 (65) | 5 | BOOT | 1.0 | 1.0 | 1.0 | NA |
| *Senegalia interior* | 63 | 52 (83) | 5 | BOOT | 1.0 | 1.0 | 1.0 | NA |
| *Styphnolobium burseroides* | 16 | 9 (56) | 5 | BOOT | 1.0 | 1.0 | 1.0 | NA |
| **Fouquieriaceae** |  |  |  |  |  |  |  |  |
| *Fouquieria leonilae* | 54 | 46 (85) | 8 | BOOT | 1.0 | 1.0 | 1.0 | 1.0 |
| *Fouquieria ochoterenae* | 33 | 21 (64) | 6 | BOOT | 1.0 | 1.0 | 1.0 | 1.0 |
| **Iridaceae** |  |  |  |  |  |  |  |  |
| *Larentia mexicana* | 26 | 20 (77) | 6 | BOOT | 0.9 | 0.8 | 0.8 | 1.0 |
| **Lamiaceae** |  |  |  |  |  |  |  |  |
| *Salvia mazatlanensis* | 37 | 24 (65) | 15 | BOOT | 1.0 | 0.5 | 0.5 | 0.8 |
| **Loranthaceae** |  |  |  |  |  |  |  |  |
| *Phthirusa inorna* | 41 | 27 (66) | 14 | BOOT | 1.0 | 1.0 | 1.0 | 1.0 |
| **Lythraceae** |  |  |  |  |  |  |  |  |
| *Cuphea glossostoma* | 29 | 19 (66) | 12 | BOOT | 0.8 | 0.5 | 0.5 | 1.0 |
| *Cuphea vesiculigera* | 51 | 31 (61) | 5 | BOOT | 0.5 | 0.5 | 0.5 | NA |
| *Cuphea viscosa* | 26 | 19 (73) | 6 | BOOT | 1.0 | 1.0 | 1.0 | 1.0 |
| **Malpighiaceae** |  |  |  |  |  |  |  |  |
| *Bunchosia sonorensis* | 49 | 46 (94) | 10 | BOOT | 1.0 | 1.0 | 1.0 | 1.0 |
| *Callaeum coactum* | 41 | 37 (90) | 18 | BOOT | 1.0 | 0.9 | 0.9 | 0.8 |
| *Galphimia vestita* | 14 | 7 (50) | 5 | BOOT | 1.0 | 1.0 | 1.0 | NA |
| *Gaudichaudia diandra* | 37 | 25 (68) | 12 | BOOT | 1.0 | 0.9 | 0.9 | 0.7 |
| *Gaudichaudia mcvaughii* | 99 | 83 (84) | 16 | BOOT | 0.9 | 0.5 | 0.5 | 1.0 |
| *Heteropterys palmeri* | 213 | 175 (82) | 6 | BOOT | 1.0 | 1.0 | 1.0 | 1.0 |
| *Lasiocarpus salicifolius* | 77 | 47 (61) | 6 | BOOT | 1.0 | 1.0 | 1.0 | 1.0 |
| **Malvaceae** |  |  |  |  |  |  |  |  |
| *Abutilon mucronatum* | 89 | 83 (93) | 33 | KFOLD | 1.0 | 0.7 | 0.7 | 0.3 |
| *Anoda paniculata* | 64 | 59 (92) | 5 | BOOT | 0.8 | 0.3 | 0.3 | 0.5 |
| *Ayenia paniculata* | 20 | 16 (80) | 5 | BOOT | 1.0 | 1.0 | 1.0 | NA |
| *Bastardiastrum gracile* | 47 | 27 (57) | 9 | BOOT | 1.0 | 1.0 | 1.0 | 1.0 |
| *Bastardiastrum incanum* | 23 | 20 (87) | 5 | BOOT | 1.0 | 1.0 | 1.0 | NA |
| *Gossypium aridum* | 358 | 269 (75) | 65 | KFOLD | 1.0 | 0.9 | 0.9 | 0.1 |
| *Hibiscus citrinus* | 44 | 36 (82) | 13 | BOOT | 0.9 | 0.9 | 0.9 | 0.6 |
| *Hibiscus jaliscensis* | 18 | 11 (61) | 6 | BOOT | 0.8 | 0.5 | 0.5 | 1.0 |
| *Horsfordia exalata* | 14 | 14 (100) | 6 | BOOT | 1.0 | 1.0 | 1.0 | 1.0 |
| *Melochia tomentella* | 46 | 38 (83) | 14 | BOOT | 1.0 | 1.0 | 1.0 | 1.0 |
| *Pavonia arachnoidea* | 22 | 18 (82) | 7 | BOOT | 1.0 | 1.0 | 1.0 | 1.0 |
| *Pavonia oxyphylla* | 62 | 38 (61) | 20 | KFOLD | 0.9 | 0.5 | 0.5 | 1.0 |
| *Physodium adenodes* | 175 | 95 (54) | 5 | BOOT | 1.0 | 1.0 | 1.0 | NA |
| *Triumfetta mexiae* | 33 | 19 (58) | 9 | BOOT | 1.0 | 1.0 | 1.0 | 1.0 |
| *Waltheria pringlei* | 151 | 130 (86) | 66 | KFOLD | 1.0 | 0.8 | 0.8 | 0.4 |
| *Waltheria tridentata* | 49 | 38 (78) | 16 | BOOT | 1.0 | 1.0 | 1.0 | 1.0 |
| **Menispermaceae** |  |  |  |  |  |  |  |  |
| *Disciphania mexicana* | 33 | 22 (67) | 8 | BOOT | 1.0 | 1.0 | 1.0 | 1.0 |
| **Nyctaginaceae** |  |  |  |  |  |  |  |  |
| *Mirabilis gracilis* | 28 | 14 (50) | 7 | BOOT | 1.0 | 1.0 | 1.0 | 1.0 |
| *Mirabilis pringlei* | 63 | 58 (92) | 18 | BOOT | 1.0 | 0.9 | 0.9 | 0.7 |
| *Salpianthus macrodontus* | 134 | 91 (66) | 8 | BOOT | 1.0 | 1.0 | 1.0 | 1.0 |
| **Orchidaceae** |  |  |  |  |  |  |  |  |
| *Aulosepalum tenuiflorum* | 25 | 18 (72) | 6 | BOOT | 1.0 | 1.0 | 1.0 | 1.0 |
| *Clowesia thylaciochila* | 11 | 6 (55) | 5 | BOOT | 1.0 | 1.0 | 1.0 | NA |
| *Erycina echinata* | 55 | 32 (58) | 10 | BOOT | 1.0 | 1.0 | 1.0 | 1.0 |
| *Mesadenus tenuissimus* | 12 | 9 (75) | 5 | BOOT | 1.0 | 1.0 | 1.0 | NA |
| **Passifloraceae** |  |  |  |  |  |  |  |  |
| *Passiflora juliana* | 95 | 91 (96) | 14 | BOOT | 0.8 | 0.8 | 0.8 | 0.6 |
| **Phyllanthaceae** |  |  |  |  |  |  |  |  |
| *Phyllanthus hexadactylus* | 14 | 12 (86) | 6 | BOOT | 0.8 | 0.5 | 0.5 | 0.8 |
| **Poaceae** |  |  |  |  |  |  |  |  |
| *Hilaria hintonii* | 14 | 11 (79) | 8 | BOOT | 0.6 | 0.5 | 0.5 | 1.0 |
| *Paspalum palmeri* | 16 | 12 (75) | 8 | BOOT | 1.0 | 1.0 | 1.0 | 1.0 |
| **Polygonaceae** |  |  |  |  |  |  |  |  |
| *Ruprechtia standleyana* | 28 | 19 (66) | 11 | BOOT | 1.0 | 1.0 | 1.0 | 1.0 |
| **Primulaceae** |  |  |  |  |  |  |  |  |
| *Bonellia pringlei* | 53 | 47 (89) | 10 | BOOT | 0.9 | 0.5 | 0.5 | 0.8 |
| **Rhamnaceae** |  |  |  |  |  |  |  |  |
| *Karwinskia johnstonii* | 64 | 64 (100) | 15 | BOOT | 1.0 | 1.0 | 1.0 | 1.0 |
| *Karwinskia rzedowskii* | 71 | 43 (61) | 8 | BOOT | 1.0 | 1.0 | 1.0 | 1.0 |
| *Karwinskia umbellata* | 71 | 63 (89) | 7 | BOOT | 0.9 | 0.5 | 0.5 | 1.0 |
| **Rubiaceae** |  |  |  |  |  |  |  |  |
| *Crusea psyllioides* | 145 | 99 (66) | 11 | BOOT | 1.0 | 1.0 | 1.0 | 1.0 |
| *Simira mexicana* | 62 | 48 (77) | 14 | BOOT | 1.0 | 1.0 | 1.0 | 1.0 |
| **Rutaceae** |  |  |  |  |  |  |  |  |
| *Amyris purpusii* | 17 | 10 (59) | 5 | BOOT | 1.0 | 1.0 | 1.0 | NA |
| *Esenbeckia hartmanii* | 77 | 40 (52) | 7 | BOOT | 1.0 | 1.0 | 1.0 | 1.0 |
| *Helietta lottiae* | 42 | 38 (90) | 14 | BOOT | 1.0 | 0.5 | 0.5 | NA |
| **Simaroubaceae** |  |  |  |  |  |  |  |  |
| *Castela retusa* | 41 | 37 (90) | 16 | BOOT | 1.0 | 1.0 | 1.0 | 1.0 |
| **Solanaceae** |  |  |  |  |  |  |  |  |
| *Datura kymatocarpa* | 70 | 67 (90) | 19 | BOOT | 1.0 | 0.5 | 0.5 | 1.0 |
| *Physalis ampla* | 51 | 30 (59) | 7 | BOOT | 1.0 | 1.0 | 1.0 | 1.0 |
| *Solanum axillifolium* | 17 | 9 (53) | 5 | BOOT | 1.0 | 1.0 | 1.0 | NA |
| *Solanum lumholtzianum* | 49 | 27 (55) | 9 | BOOT | 1.0 | 1.0 | 1.0 | 1.0 |
| *Solanum pinnatisectum* | 339 | 172 (51) | 21 | KFOLD | 1.0 | 1.0 | 1.0 | 1.0 |
| **Surianaceae** |  |  |  |  |  |  |  |  |
| *Recchia connaroides* | 31 | 22 (71) | 6 | BOOT | 1.0 | 0.5 | 0.5 | 1.0 |
| **Verbenaceae** |  |  |  |  |  |  |  |  |
| *Bouchea dissecta* | 119 | 87 (73) | 29 | KFOLD | 1.0 | 0.9 | 0.9 | 0.4 |
| *Lippia mcvaughii* | 39 | 33 (85) | 5 | BOOT | 1.0 | 1.0 | 1.0 | NA |
| **Violaceae** |  |  |  |  |  |  |  |  |
| *Hybanthus serrulatus* | 24 | 13 (54) | 7 | BOOT | 0.9 | 0.5 | 0.5 | 1.0 |
| **Ximeniaceae** |  |  |  |  |  |  |  |  |
| *Ximenia pubescens* | 40 | 28 (70) | 10 | BOOT | 1 | 1 | 1 | 1 |
