# Supplementary material for: Endemic vascular plants provide reliable indicators for mapping seasonally dry tropical forests
Source: PLoS One. 2025 Dec 9;20(12):e0337886. doi: 10.1371/journal.pone.0337886 (PMC12688124; doi:10.1371/journal.pone.0337886)
Supplement: Attachment — Submitted filename: Review Plos Maxico.pdf [file pone.0337886.s004.pdf]

The paper entitled; "**Are endemic species a suitable surrogate for delineating the geographical distribution of the tropical dry forest?**" is based on the original and innovative approach. The paper is technically sounds and is fully supported by the reviewed data on scientific bases but needs some improvement.

This paper will add significant approached to the field of advances in plant ecology. This article is based on importance of endemic species by using the occurrence records of species obtained from the Global Biodiversity Information Facility (GBIF) database and different software in R-Package. The study examines the seasonally dry tropical forest (SDTF) in Mexico, based on the distribution area of Mexican endemic vascular plant species registered exclusively in this biome. However, the authors must check the entire manuscript for the typographical mistakes, grammar mistake, spelling mistakes and incorporate some suggestions that might improve the manuscript. The language of the manuscript should be improved significantly, and the spelling/grammatical mistakes should be eliminated. The key findings of this study must be properly elaborated to draw solid conclusions.

General comments of this article are:

Query No. 1: Aims and objectives part of abstract are not clear. It is important to highlight the purpose of study. It should be added in the abstract and linked with title The abstract should be according to the journal format i.e. Back ground, results and conclusions. The following questions must be answer logically in the abstract and manuscript:

- Why you selected endemic species as a surrogate for tropical dry forest boundaries?
- What are the advantages of adopting this approach in applied ecology?
- Can the presence of endemic species reliably indicate ecological integrity or habitat continuity in tropical dry forests? Why or why not?

The **keywords** need revision to be arranged alphabetically and must be in accordance to abstract.

Query No. 2. In introduction, Authors should add background justification related to their study project and research hypothesis with sound evidences supported by litature review.

Query No. 3. What is need of the present study?? Authors should add 1 paragraph about Gape analysis and importance of present study.

Query No. 4. Why the authors choose of data would be necessary to test whether endemic species accurately reflect the extent of tropical dry forests? To what extent your results are accurate and acceptable.

Query No. 5. Why you selected ecological models you selected the present study in field of plant Sciences and others???

Query No. 6. Should endemic species be used alone, or in combination with other indicators (e.g., climate, soil, vegetation structure)? Why?

Query No. 7. Discussion and Conclusion needs improvement. What are the key findings of the study and suggest future recommendations?

Query No. 8. Bridge a link between results and Discussion. This is suggested to discussed your results appropriately in the context of previous available literature to highlight the novelty of this study.

Query No. 9. Conclusion should be recompiled with sound concluding remarks and key findings with future recommendations. I suggest that this article should cover the following questions:

- How does the use of endemic species approach could be compare with other biogeographic surrogate methods, such as remote sensing or ecoregion mapping? Support your methodology used or approach adopted in this study with solid justification.
- What case studies either with in Mexico, or other parts of the world explicate the failure or success stories of using endemic species as surrogates for dry forest delineation?
  Which is very important tool for future conservation strategies and biodiversity protection.

Query No. 10. What is the novelty of yours research and how this would be applied on broader scale, especially for biodiversity conservation?

Query No. 11. Cross check all the references cited in the entire article, all the references must be in uniform language e.g. Ref. no. 39.

Rabia Afza (PhD)
Department of Botany,
Hazara University Mansehra, Pakistan
rabiaafza@hu.edu.pk/ethnopk@gmail.com
