# Supplementary material for: Endemic vascular plants provide reliable indicators for mapping seasonally dry tropical forests
Source: PLoS One. 2025 Dec 9;20(12):e0337886. doi: 10.1371/journal.pone.0337886 (PMC12688124; doi:10.1371/journal.pone.0337886)
Supplement: Attachment — Submitted filename: Response to reviewers_11-03-25.docx [file pone.0337886.s005.docx]

**Manuscript title**: *Endemic vascular plants provide reliable indicators for mapping Seasonally Dry Tropical Forests*

**Manuscript ID**: PONE-D-25-28829
**Corresponding Author**: Dr. Guillermo Ibarra-Manríquez

**Reviewer #1:** Manuscript: Are endemic species a suitable surrogate for delineating the geographical distribution of the tropical dry forest

**GENERAL COMMENTS:**

- **This is a methodologically robust and well-documented study that applies ecological niche modeling and species distribution models (SDMs) to evaluate the efficacy of endemic species in delineating the Seasonally Dry Tropical Forest (SDTF) in Mexico. Selecting 50%, 75%, and 100% thresholds is only briefly explained. Why these specific thresholds?**

**Authors’ Response:** The thresholds of 50%, 75%, and 100% were chosen to categorize species according to their degree of association with the SDTF, following the Majority method (Murphy et al., 2019). Specifically, the 50% threshold defines characteristic species, the 75% threshold identifies high-affinity species, and the 100% threshold corresponds to strict endemics. This hierarchical classification ensures that each species is associated with a single dominant region, providing a robust basis for subsequent ecological niche modeling. In this sense, our approach enhances comparability with other regionalization studies (e.g., Moonlight et al., 2020; Murphy & Smith, 2021). We have now clarified this rationale in the new version of the manuscript **(see Methods section, lines 157-168).**

- **Add a brief justification or reference for these cutoffs, are they standard in the literature, or did exploratory analysis suggest these were optimal?**

**Authors’ Response:** The thresholds of 50%, 75%, and 100% follow the Majority method standard in regionalization analyses and have been applied in other biogeographic frameworks to distinguish species with varying levels of ecological fidelity (Murphy et al., 2019). As we said, the 50% cutoff ensures that a species is primarily associated with a single dominant region, while 75% and 100% represent increasingly strict criteria for identifying high-affinity and endemic taxa, respectively. This hierarchical classification facilitates robust comparative analyses among taxa and regions (see Kreft & Jetz, 2010; Moonlight et al., 2020).

- **Why 5 km was chosen specifically for SDTF species.**

**Authors’ Response:** The selection of a 5 km threshold for defining the spatial independence of records in the SDTF follows the recommendations of Graham et al. (2008), who demonstrated that georeferencing uncertainties can significantly influence model outcomes. In this sense, a 5 km buffer provides an appropriate balance between capturing environmental variability and minimizing spatial errors in species occurrence data. Similar spatial resolutions have been successfully applied in SDM studies of tropical dry forests and other heterogeneous landscapes. We have now clarified this rationale in the new version of the manuscript **(see Methods section, lines 175-177).**

- **Please clarify? Lowest Presence Threshold (LPT) is acceptable for this study?**

**Authors’ Response:** Yes, the LPT is appropriate for this study. Applying the LPT ensures that a high proportion of occurrence records are retained in the binary model while minimizing omission errors, particularly for species with limited data. It is widely accepted for small-sample SDMs (Pearson et al., 2007; Merow et al., 2013; Muscatello et al., 2021) and accurately represents species’ potential distributions under data constraints. We have now clarified this rationale in the new version of the manuscript **(see Methods section, lines 186-189).**

- **If possible please add “AUC values in Supplementary Material to allow quick assessment of model quality.**

**Authors’ Response:** We have added the AUC values to the Supplementary Material to facilitate rapid assessment of model quality. Additionally, we incorporated three complementary evaluation metrics — sensitivity, specificity, and the True Skill Statistic (TSS)—to provide a more comprehensive evaluation of model performance (Márcia-Barbosa et al., 2013; Muscatello et al., 2021).

- **Reference style is very odd, just copied in different styles????**

**Authors’ Response:** We have thoroughly revised the reference list to ensure they follow a consistent style in accordance with *Plos ONE* guidelines.

**MAJOR COMMENTS**

- **Title is good and satisfactory**

No response required.

- **Abstract missing some points like conclusion and recommendation in last few lines. I recommend thoroughly reviewing relevant literature and preparing a comprehensive manuscript, as it will represent our work at an international level**

**Authors’ Response:** The abstract has been revised to include clear concluding statements and concise recommendations. Although our study focuses on Mexico, the proposed framework is broadly applicable to other regions with similar ecological contexts. These revisions enhance the international relevance of our work and clarify its implications for biodiversity conservation and the delineation of biomes.

- **Introduction. No sequence in the introduction sections, some paragraphs are very brief, some are too long, references needs to be updated, data is scattered and many phrases are repeated, please correct.**

**Authors’ Response:** The Introduction section has been thoroughly reorganized to improve logical flow and readability. Paragraphs were adjusted for balance and coherence, outdated citations were replaced with recent and relevant literature (e.g., Ibarra-Manríquez et al., 2022; Ortiz & Villaseñor, 2025; Murphy & Smith, 2021), and redundant phrases were removed. These revisions provide a clearer conceptual framework and strengthen the theoretical foundation of the study.

- **Results. You mention overprediction briefly, but underlying causes (e.g., data quality, generalist species) are not thoroughly explored.**

**Authors’ Response:** The Results and Discussion sections have been expanded to include a more detailed discussion of the potential causes of overprediction, such as the inclusion of generalist species and variability in data quality. These explanations are supported by recent studies addressing SDM accuracy and uncertainty in heterogeneous environments (Franklin, 2013; Muscatello et al., 2021). **(see Discussion section, lines 342-355).**

- **Discussion. Add a paragraph discussing how model bias or poor environmental variable resolution may influence overprediction.**

**Authors’ Response:** A new paragraph has been added to the *Discussion* to examine how model bias and the spatial resolution of environmental predictors can influence overprediction **(see Discussion section, lines 383-390).**

- **Discuss broader applications and limitations of your approach in other dry forest systems.**

**Authors’ Response:** We have expanded the Discussion section to address the broader applicability and limitations of our framework across other tropical dry forest systems worldwide. This section now emphasizes the value of combining endemic-species data with environmental predictors for comparative and large-scale biodiversity assessments (e.g., Dexter et al., 2018; Pennington et al., 2018). **(see Discussion section, lines 383-390).**

- **Conclusion. Key findings are missing? Limitations, practical implications, future research should be clearly mentioned**

**Authors’ Response:** The Conclusions section has been revised to include explicit statements of the study’s key findings, methodological limitations, and practical implications for conservation planning. We also added suggestions for future research directions, including the integration of phylogenetic and functional diversity measures **(see Conclusions, lines 401-414)**.

- **Overall, the manuscript should require major revision to be published in a reputed Journal like PLOS ONE**

**Authors’ Response:** All points requested by the reviewers were addressed with the intention of meeting the demanding quality criteria of PLOS ONE. The revised version incorporates updated references, clearer argumentation, and improved methodological transparency, which together strengthen both the conceptual and applied contributions of the manuscript.

**Reviewer #2:** The paper entitled; “Are endemic species a suitable surrogate for delineating the geographical distribution of the tropical dry forest?” is based on the original and innovative approach. The paper is technically sound and is fully supported by the reviewed data on scientific bases, but needs some improvement. This paper will add significant approached to the field of advances in plant ecology.

**GENERAL COMMENTS:**

- **This article is based on importance of endemic species by using the occurrence records of species obtained from the Global Biodiversity Information Facility (GBIF) database and different software in R-Package. The study examines the seasonally dry tropical forest (SDTF) in Mexico, based on the distribution area of Mexican endemic vascular plant species registered exclusively in this biome. However, the authors must check the entire manuscript for the typographical mistakes, grammar mistake, spelling mistakes and incorporate some suggestions that might improve the manuscript. The language of the manuscript should be improved significantly, and the spelling/grammatical mistakes should be eliminated. The key findings of this study must be properly elaborated to draw solid conclusions.**

**Authors’ Response:** The entire text has been thoroughly revised for language accuracy and clarity, and the key findings have been more clearly stated in the Results and Conclusions to strengthen the manuscript’s overall quality.

- **Query No. 1: Aims and objectives part of abstract are not clear. It is important to highlight the purpose of study. It should be added in the abstract and linked with title The abstract should be according to the journal format i.e. Background, results and conclusions. The following questions must be answer logically in the abstract and manuscript:**

**Authors’ Response:** The abstract has been thoroughly rewritten to follow the *PLOS ONE* structured format. The main objective and purpose of the study are now explicitly stated and directly linked to the title. These revisions improve readability and clarify the broader significance of our approach.

1. ***Why did we select endemic species as a surrogate for delineating tropical dry forest boundaries?***

**Authors’ Response:** Endemic species were selected because they exhibit high ecological fidelity and restricted geographic distributions, making them reliable indicators of specific biomes. Their narrow ranges and strong associations with environmental stability allow them to represent the ecological and spatial limits of the Seasonally Dry Tropical Forest (Prieto-Torres & Rojas-Soto, 2016; Sosa et al., 2018). We have added a clarifying statement to explain this point in the new version of the manuscript (see the **Introduction section, lines 129-136**).

2. ***What are the advantages of this approach in applied ecology?***

**Authors’ Response:** Using endemic species as surrogates offers a practical and replicable tool for applied ecology. This approach enables spatially explicit frameworks that support biodiversity conservation, identify priority areas, and assess connectivity in fragmented landscapes (see Dexter et al., 2018).

3. ***Can their presence reliably indicate ecological integrity or habitat continuity? Why or why not?***

**Authors’ Response:** Yes. The presence of endemic species reliably indicates ecological integrity and habitat continuity because these taxa are strongly tied to stable climatic, edaphic, and biotic conditions. Their persistence in specific areas reflects long-term environmental stability and helps identify natural biome boundaries (Pennington et al., 2009; DRYFLOR, 2016).

4. ***The keywords need revision to be arranged alphabetically and must be in accordance to abstract.***

**Authors’ Response:** The keywords have been revised to follow alphabetical order and to match the terminology used in the abstract: *Biome delineation; Endemic species; High-affinity species; Species distribution modeling; models ensample.*

- **Query No. 2. In introduction, Authors should add background justification related to their study project and research hypothesis with sound evidences supported by literature review.**

**Authors’ Response:** We have revised the Introduction to include a clearer theoretical background and justification for the study, supported by up-to-date references (e.g., Ibarra-Manríquez et al., 2022; Ortiz & Villaseñor, 2025; Murphy & Smith, 2021). The revised section now explicitly states the research hypothesis and situates it within current debates on biome delineation and tropical dry forest ecology.

- **Query No. 3. What is need of the present study?? Authors should add 1 paragraph about Gap analysis and importance of present study.**

**Authors’ Response:** In response, we have added a new paragraph in the Introduction section (**see Lines 129–136**). The new text emphasizes the necessity for species-based, spatially explicit approaches to define the SDTF, thereby addressing critical gaps in traditional mapping and regionalization efforts (Moonlight et al., 2020; Murphy & Smith, 2021). This addition clarifies the novelty and importance of our contribution to biodiversity conservation and biogeography.

- **Query No. 4. Why the authors choose of data would be necessary to test whether endemic species accurately reflect the extent of tropical dry forests? To what extent your results are accurate and acceptable.**

**Authors’ Response:** The data selection was guided by our objective to test whether endemic species accurately represent the geographic extent of the SDTF. Endemic taxa are confined to specific ecological conditions, making them effective biogeographic indicators (Prieto-Torres & Rojas-Soto, 2016; Sosa et al., 2018). Our results showed high predictive accuracy (96%) for the modeled species, validated through Asteraceae occurrence data and confusion matrix analyses. These steps confirm the robustness and reliability of our approach, ensuring its applicability to other biogeographic systems (Franklin, 2013; Moonlight et al., 2020).

- **Query No. 5. Why you selected ecological models you selected the present study in field of plant Sciences and others???**

**Authors’ Response:** Species distribution models (SDMs) were selected because they are widely used and theoretically grounded tools for inferring species–environment relationships (Guisan & Zimmermann, 2000; Franklin, 2013). Given the geographic extent of our study and the limited availability of field data, species distribution models (SDM) offers an efficient, objective, and replicable method for delineating biomes. This framework allows comparison among species with different levels of affinity to the biome, ensuring scalability to other ecosystems (Murphy & Smith, 2021; Moonlight et al., 2020). We added a clarifying statement explaining this point in the new version of the manuscript **(see Introduction section, lines 78-97).**

- **Query No. 6. Should endemic species be used alone, or in combination with other indicators (e.g., climate, soil, vegetation structure)? Why?**

**Authors’ Response:** While endemic species are valuable indicators of biome identity due to their strong associations with specific ecological conditions, they do not capture all dimensions of ecosystem complexity. In our study, the ecological niche models already incorporate climatic and edaphic variables (see Supplementary Material), thereby integrating key abiotic factors that influence species distributions.

This approach combines the biological information provided by endemic species with relevant environmental gradients. The present study specifically tested the effectiveness of endemics as standalone indicators of the SDTF; however, future research could compare biome delineations derived from endemic taxa with those based on the entire flora or on multi-indicator approaches to provide deeper insights into the relative contributions of different species groups to biome boundaries. Such comparative analyses could further refine multi-indicator frameworks for biome mapping and conservation prioritization (Dexter et al., 2018)

- **Query No. 7. Discussion and Conclusion needs improvement. What are the key findings of the study and suggest future recommendations? results appropriately in the context of previous available literature to highlight the novelty of this**

**Authors’ Response:** We have revised both the Discussion and Conclusions to highlight key findings, situate them within the broader literature, and underscore the novelty of our approach (Moonlight et al., 2020; Murphy & Smith, 2021). Future research directions and conservation implications are now explicitly included **(see Conclusions section, lines 401-416).**

- **Query No. 8. Bridge a link between results and Discussion. This is suggested to discussed your study.**

**Authors’ Response:** In response, the Discussion section has been modified to explicitly connect our Results with their ecological and biogeographic implications. We now emphasize how the strong predictive performance of high-affinity endemic species supports their use as surrogates for biome delineation, consistent with previous work on vegetation mapping and ecoregion frameworks.

- **Query No. 9. Conclusion should be recompiled with sound concluding remarks and key findings with future recommendations. I suggest that this article should cover the following questions:**

1. ***How does the use of endemic species approach could be compare with other biogeographic surrogate methods, such as remote sensing or ecoregion mapping?***

**Authors’ Response:** Our findings demonstrate that high-affinity endemic species provide a biologically meaningful and spatially precise delineation of biomes compared to remote sensing or ecoregion mapping, which may overlook ecological boundaries driven by species turnover (Franklin, 2013; Moonlight et al., 2020).

2. ***Support your methodology used or approach adopted in this study with solid justification.***

**Authors’ Response:** Endemic species occupy narrow environmental niches that mirror the ecological parameters defining biomes. Integrating ENM with these affinities provides a robust, data-driven framework supported by high predictive accuracy and independent validation (Pearson et al., 2007; Merow et al., 2013). This approach is further supported by its high predictive accuracy (96%), validated through independent datasets (Asteraceae occurrence records) and confusion matrix analyses.

3. ***What case studies either with in Mexico, or other parts of the world explicate the failure or success stories of using endemic species as surrogates for dry forest delineation?***

**Authors’ Response:** Previous research in Mexico and other regions (e.g., South America, Madagascar) has shown that endemics can effectively delineate ecosystems with high evolutionary significance, whereas failures occur when environmental gradients are ignored (Sosa et al., 2018; DRYFLOR, 2016). Our approach addresses these limitations by integrating endemic species distributions with climatic and edaphic variables, ensuring a more accurate and ecologically grounded delineation of the SDTF.

4. ***Which is very important tool for future conservation strategies and biodiversity protection.***

**Authors’ Response:** We conclude that endemic-species data with ENMs provides a powerful tool for conservation and biodiversity management. We recommend incorporating beta diversity and phylogenetic turnover in future analyses to refine this framework and apply it to other biomes, thereby improving systematic conservation planning and restoration strategies. To further enhance its applicability, we recommend incorporating beta-diversity metrics, phylogenetic turnover, and full-flora analyses in future studies to validate and refine the surrogate value of endemics. Expanding this integrative approach to other biomes will strengthen systematic conservation planning, promote efficient resource allocation, and improve the long-term management of biodiversity hotspots worldwide.

- **Query No. 10. What is the novelty of yours research and how this would be applied on broader scale, especially for biodiversity conservation?**

**Authors’ Response:** The novelty of this research lies in demonstrating that endemic species with high ecological affinity can serve as effective biogeographic surrogates, yielding more precise and evolutionary meaningful biome boundaries than traditional mapping methods. By integrating ecological niche modeling with environmental affinity data, we establish a replicable, data-driven framework applicable to other biodiversity-rich biomes (Moonlight et al., 2020; Murphy & Smith, 2021). This approach informs conservation planning by identifying priority areas and anticipating species responses to climate change, thereby bridging biogeographic theory with applied conservation practice and providing a transferable tool for global biodiversity assessments.

- **Query No. 11. Cross check all the references cited in the entire article, all the references must be in uniform language e.g. Ref. no. 39.**

**Authors’ Response:** All references have been thoroughly checked for consistency and reformatted to comply with the *PLOS ONE* reference style. Citation order and numbering have been standardized throughout the text and reference list.

**REFERENCES CITED DURING THIS REVIEW**

Dexter, K. G., Pennington, R. T., Oliveira-Filho, A. T., Bueno, M. L., Silva de Miranda, P. L., & Neves, D. M. (2018). Inserting tropical dry forests into the discussion on biome transitions in the tropics. *Frontiers in Ecology and Evolution*, *6*, 104.

Kreft, H., & Jetz, W. (2010). A framework for delineating biogeographical regions based on species distributions. *Journal of Biogeography*, *37*(11), 2029-2053.

Márcia Barbosa, A., Real, R., Muñoz, A. R., & Brown, J. A. (2013). New measures for assessing model equilibrium and prediction mismatch in species distribution models. *Diversity and Distributions*, *19*(10), 1333-1338.

Merow, C., Smith, M. J., & Silander Jr, J. A. (2013). A practical guide to MaxEnt for modeling species' distributions: what it does, and why inputs and settings matter. *Ecography*, *36*(10), 1058-1069.

Moonlight, P. W., Silva de Miranda, P. L., Cardoso, D., Dexter, K. G., Oliveira‐Filho, A. T., Pennington, R. T., ... & Särkinen, T. E. (2020). The strengths and weaknesses of species distribution models in biome delimitation. *Global Ecology and Biogeography*, *29*(10), 1770-1784.

Murphy, D. J., Ebach, M. C., Miller, J. T., Laffan, S. W., Cassis, G., Ung, V., ... & Tursky, M. L. (2019). Do phytogeographic patterns reveal biomes or biotic regions?. *Cladistics*, *35*(6), 654-670.

Murphy, S. J., & Smith, A. B. (2021). What can community ecologists learn from species distribution models?. *Ecosphere*, *12*(12), e03864.

Muscatello A et al. 2021. *How decisions about fitting species distribution models affect conservation outcomes.* Conserv. Biol. 35: 1309–1320.

Pearson, R. G., Raxworthy, C. J., Nakamura, M., & Townsend Peterson, A. (2007). Predicting species distributions from small numbers of occurrence records: a test case using cryptic geckos in Madagascar. *Journal of biogeography*, *34*(1), 102-117.

Pennington, R. T., Lehmann, C. E., & Rowland, L. M. (2018). Tropical savannas and dry forests. *Current Biology*, *28*(9), R541-R545.
